# Supplementary material for: Are carbon emissions trading and green financial instruments synergistic? -Comprehensive quantitative research based on content analysis
Source: PLoS One. 2024 Mar 7;19(3):e0298601. doi: 10.1371/journal.pone.0298601 (PMC10919601; doi:10.1371/journal.pone.0298601)
Supplement: S1 Data — (ZIP) [file pone.0298601.s001.zip › Data/地级市Z_F工作报告.zip!/─┌├╔╣┼/░n═╖╩╨/└┤╫╘╣1⁄2╓┌║┼í╛┬φ┐╦╩2╛▌═°í┐_2015.docx]

各位代表：

　　现在，我代表市政府向大会报告工作，请予审议，并请市政协各位委员提出意见。

　　2014年工作回顾

　　2014年是令人难忘的。面对严峻的经济形势和繁重的改革任务，全市各族人民在市委的坚强领导下，认真贯彻落实习近平总书记系列重要讲话精神和自治区“8337”发展思路，主动应对挑战，奋力攻坚克难，啃下了不少硬骨头，经济社会发展和人民生活水平有了新的提高。预计地区生产总值3700亿元，增长8.5%;固定资产投资3440亿元，增长15%;社会消费品零售总额1180亿元，增长10%;公共财政预算收入234.3亿元，增长8.9%;城乡居民人均可支配收入分别达到35640元和12820元，增长9%和11%。

　　一、全面加强经济调控

　　一是着力稳工业。去年初，煤电油运等先行指标均呈负增长，主要工业产品量价齐跌，规模以上企业亏损面达到31%，94户企业处于停产半停产状态。为此，我们在自治区率先出台促进工业经济平稳健康发展的八条扶持政策，共为企业争取产业扶持资金6.5亿元、电价补贴7.9亿元，协调落实贷款176亿元，市本级投入贷款贴息等扶持资金10亿元，减免企业税收2.4亿元，有力扭转了工业下滑局面。截至年底，71户企业扭亏为盈，70%的停产企业恢复生产。预计全年规模以上工业增速11%，在自治区排名第2位。

　　二是着力调结构。围绕破解工业围城，确立“东铝、西钢、南高、北装”发展布局，重点打造四个千亿园区。东西两园区抓产业转型升级和淘汰落后产能，电解铝和稀土钢深加工占比分别提高7.3和5个百分点。南北两园区抓产城一体，北建生态屏障，南留发展空间，高新技术产业和装备制造业占比分别提高3.9和2个百分点。全市单位GDP能耗下降4.5%，提前一年完成“十二五”目标任务，包头获批国家节能减排财政政策综合示范城市。围绕市委“5421”战略定位，制定新型冶金、装备制造等15个发展规划，出台发展非公经济、县域经济的实施意见，推动工业转型升级、服务业提质增量、农牧业集约高效。装备制造、钢铁、铝业增速分别达到14%、12%和19%，新能源、稀土新材料等战略新兴产业增长13%，规模以上非公企业占全市工业比重达到65.2%。服务业投资增长20%，企业注册数量增长61.5%。农牧业“菜薯肉乳”四大主导产业产值占比达70%以上。围绕城乡统筹优化区域布局，推动市五区组团式布局、一体化发展，东河区、石拐新区面貌显著改观。推动山北大型清洁能源基地、原材料基地和土右旗能源重化工基地建设，县域经济加快发展。

　　三是着力扩投资。组织实施亿元以上重点项目583个，完成投资1577亿元。包钢稀土板材等233个项目竣工投产，神华煤制烯烃二期等281个项目开工建设，华电土右电厂、国储能煤基多联产等项目获得国家、自治区核准。实施招商引资项目855个，引进资金640亿元，增长20%，深圳比亚迪新能源矿用车、广东鸿达兴业稀土助剂等一批高新项目签约。加大政府主导投资力度，基础设施和生态建设投入785亿元，拉动全市投资增长5个百分点。

　　四是着力促消费。面对大宗商品消费下滑，传统服务业增长低迷的局面，及时出台支持服务业发展的八条措施，刺激消费需求。推动银企对接，为企业争取协议贷款额度2281亿元，辖区内银行业贷款余额达到1834亿元，增长12.6%。在国内主要媒体加大城市形象宣传力度，旅游业总收入预计达到256亿元，增长23%。成功举办首届国际装备制造博览会和首届中国(国际)牛羊肉产业大会等21场大型会展活动，实现交易额80亿元。房交会成交商品房8056套，交易额达到54亿元。加快发展电子商务，电商交易额达到56亿元。提升传统商贸业，实施了乌兰道商业街改造、东河维多利商厦等75个重点项目，完成投资244亿元。

　　二、加快提升城市综合发展水平

　　一是推进工业园区建设。9个工业园区共完成道路、管网、污水处理等基础设施投资21亿元，一批企业和项目入园发展，产业集中度提高4.8个百分点。稀土高新区和装备制造园区营业收入分别达到1360亿元和1100亿元，金属深加工园区和铝业园区分别突破600亿元和500亿元，铝业园区获批国家“城市矿产”示范基地。

　　二是推进新都市区建设。成立新都市区管委会，进一步完善控制性详规，征拆房屋14.4万平方米，收储土地745亩。市民公园、市民广场和道路、综合管廊等基础设施完成投资7亿元，学校、幼儿园等公共服务设施建设有序推进，富力城、包商大厦等开发项目入驻实施，新都市区框架基本形成。

　　三是推进现代农牧业示范园区建设。出台加快现代农牧业发展的实施意见，启动建设4个现代农牧业示范园区和30个农牧业示范基地，已入驻80余家企业，增加值占全市农牧业的45%。新晋2个国家级龙头企业、9个国家级农牧民专业合作社。土右旗和九原区分别被评为国家和自治区级示范园区，达茂旗被评为全国草原保护建设科技综合示范区。

　　四是推进现代物流园区建设。确定物流业“六园区、一支点”发展布局，实施铁路现代综合物流园、土右煤炭物流园等36个重点项目，完成投资80亿元。苏宁内蒙古配送中心等15个项目建成运营，土右煤炭物流园数字交易平台上线交易量突破4000万吨。结合110、210国道改造促进物流资源整合，钢铁建材物流园区新增企业368家，集中度达到85%。

　　五是推进文化旅游景区建设。加快文化与旅游的深度融合，实施文化旅游项目33个，完成投资18.4亿元。五当召、美岱召5A级景区和希拉穆仁草原等10个4A级景区创建工作全面启动。五当召旅游公路建成通车，九峰山景区获批为敕勒川国家森林公园，成功举办第二届中国游牧文化旅游节。组织实施22个重点文化产业项目，完成投资11.5亿元，美德亨收藏文化博览中心等一批项目建成运行。

　　六是推进土右新型中等城市和达茂白云边境口岸型城市建设。全面完成撤旗设市、合并设市前期工作，已上报国务院待批。土右旗、达茂旗和白云矿区经济增速预计均在10%以上，完成基础设施投入76亿元，拓展城区面积7.4平方公里，综合实力进一步提升。面对进出口贸易下滑影响，始终抓好满都拉口岸建设，投资2.2亿元完成五进五出通道、边检大楼和仓储物流等配套设施建设。抢抓国家“一带一路”发展战略机遇，加强与蒙古国合作，口岸常年开放和跨境经济合作区项目已上报国家审批。

　　三、大力实施民心工程

　　一是推进北梁棚户区改造工程。历经2013年百日攻坚和2014年春秋两季会战，北梁棚改征拆任务全部完成，累计拆迁房屋5.36万户、430万平方米，相当于一年时间拆掉一个中等县城。建设安置房3.1万套、273万平方米，回购安置房1.2万套、85万平方米，安置居民10.9万人，北梁居民在新的家园幸福生活。包头被住建部命名为“全国棚户区改造示范城市”。

　　二是实施扶贫攻坚和“十个全覆盖”工程。认真开展“三到村三到户”精准扶贫，落实资金8.12亿元，解决了3.1万贫困人口的掉队问题。投入21.4亿元改善农村牧区生活条件，建设高标准示范村71个，达标村159个，受益人口27万。

　　三是启动大气污染防治工程。强化“治气、减煤、降尘、控车、严管”五项措施，治理污染企业344家，关停取缔大青山采石和开矿企业158家，整治煤场料场392家、城区燃煤锅炉702台，新增集中供热653万平方米，淘汰黄标车及老旧车辆1.2万辆。全市空气PM2.5浓度下降29.5%，二氧化硫平均浓度下降45%，看得见蓝天白云的好天气明显增多。

　　四是启动城市供水改造工程。按照人喝地下水、机器用黄河水的原则，封停企业自备井113眼，减少工业用地下水720万立方米，相当于城区居民4年的健康水饮水量。城区健康水新增受益人口22.6万，农村牧区安全饮水新增受益人口5.8万。实行水质监管与供水经营分离，建成检测106项指标的一流水质检测中心，居民用水安全得到保障。

　　五是启动食品安全监管工程。整合食品安全监管职能，实现生产、流通、消费环节统一监管。推进县乡村三级监管网络建设，开展食品安全专项整治行动。开工建设九原区、土右旗、青山区3个集加工、仓储、配送、检验于一体的食品加工园区。在自治区率先建立政府主导的农畜产品质量安全追溯体系，首批50个试点陆续建成运行，包头获批全国肉菜流通追溯体系试点城市。

　　六是启动交通设施改善工程。以空前力度打通青山路、哈屯高勒路、兵工路、站前路等33条城区断头路。高标准完成钢铁大街、阿尔丁大街提质改造，开辟公交专用车道，道路通行能力提高20%，公交车速提高30%。新增公交线路11条、新能源公交车144台。全面启动南绕城公路改扩建工程，新建续建5条城乡一级公路，省道211线固阳至百灵庙、省道104线百灵庙至白云鄂博和包石一级公路建成通车。包头火车站广场完成升级改造，机场新航站楼投入使用。

　　七是启动生态环境整治工程。公路铁路沿线和城市出入口累计拆除违章建筑56万平方米，绿化里程近400公里，新增绿化面积3700多公顷。完成国家林业重点工程37.5万亩，六大重点区域绿化11万亩。新建改造奥林匹克、鹿园等14个公园广场。治理昆河、四道沙河等河道22公里，完成景观蓄水10.7公里。

　　各位代表，2014年公共财政用于民生和社会事业支出达到292亿元，占公共财政预算支出82.5%。全年新增城镇就业4.1万人，城镇登记失业率控制在3.87%。企业退休人员基本养老金每人每月提高192元，城镇低保每人每月提高15元，农村牧区低保每人每年提高144元。城镇职工、居民和农牧民住院费用支付限额分别提高到38.7万元、20.1万元和13万元。筹资6120万元为11万农牧户发放了“暖心煤”。为986名低保户大学生发放资助金3567万元。建成55所公办幼儿园，学前三年入园率达到90%。新改扩建卫生服务机构122所，基层全部实行药品零差率销售。土右旗、达茂旗被评为国家级卫生县城。投资15亿元改造老旧小区268个，解决了设施老化和脏乱差问题。对昆区钢32街坊10万平方米苏式建筑进行修旧如旧改造，保留了城市历史记忆。

　　四、积极推进重点领域改革

　　一是激发经济发展活力。全面深化行政审批制度改革，市本级行政审批事项由269项减少到43项，减幅84%;市辖区保留23项，减幅70%;旗县保留27项，减幅68%。集中审批职能，优化审批流程，审批事项平均办理时限由16天压缩到4天。政府为房地产项目环评、能评、水评统一埋单，实行工业项目评估前置改后置，房地产开发和工业项目审批时限分别压缩77%和91%。市本级行政事业收费由85项减少到40项，每年为企业和个人减负6000余万元。推行工商登记“先照后证”和“三证合一”改革，工商注册登记同比增长70.2%，办理时限由15天减少到3天。成立公共资源交易中心，实现政府投资项目、国有资产交易、土地招拍挂和政府采购集中统一进场交易，取消了对民营投资强制进场交易的管制。加快土地流转制度改革，全市土地流转面积累计180万亩，土右旗土地确权登记颁证试点和达茂旗草原确权承包试点工作稳步推进。土右旗“扩权强县”试点改革第一批经济社会管理权限已下放51项。

　　二是完善现代市场体系。出台支持金融业发展和加快企业上市的指导意见，扩大企业直接融资比重，包钢集团首期30亿元和稀土高新区、职教园区14亿元企业债券成功发行，展浩电气成为自治区首家在新三板挂牌上市的中小企业。整合组建水务投资集团、交通投资集团等4个城市建设融资平台。制定出台科研资金管理办法，重点支持产学研合作项目，中科院包头稀土新材料研发中心获准设立，浙大包头工业技术研究院、中科院绿色农业包头中心、中国农大院士工作站、内蒙农科院包头分院挂牌运营，北大工学院包头研究院升级为校级研究院。实施新建项目环保准入，严禁在建成区新建高污染、高耗能、高排放项目。重金属污染物责任保险试点工作有序开展，第一批28家试点企业陆续参保。

　　三是提高社会治理能力。推行基本公共服务均等化，实现市五区、石拐区社会救助一体化和基本公共服务全覆盖。加强在编人员管理，严格整治超编、吃空饷问题，对事业单位空编实行逢进必考、择优录用。推行“划片招生、电脑派位”改革，促进中考公平公正。投资10亿元推进全市公共文化标准化、均等化发展，博物馆、群艺馆等公益单位按照法人治理结构组建理事会。全市30个街道、115个社区实施了“精街道、强社区、促服务”改革，青山区、昆区被命名为全国示范区。依法规范信访秩序，妥善化解了一批信访积案。城市视频监控系统建设有序推进，四级巡控体系更加完善。顺利通过全国文明城市第四次测评。

　　五、切实加强政府自身建设

　　一是坚持把党的群众路线教育实践活动摆在突出位置。围绕“为民务实清廉”主题，突出整治“四风”问题，实施了9个方面、128项整改措施，有效解决了一批关系群众切身利益和联系服务群众“最后一公里”的问题，干部作风明显改进，执行力落实力进一步增强，凝聚了干事创业的正能量。

　　二是加强法治政府建设。自觉接受人大、政协监督，建立定期征求意见制度，实行政府常务会议题向人大报告、向政协征求意见制度。制定实施行政审批管理规定、行政事业收费管理规定和规范行政处罚自由裁量权规定。向市人大提请地方性法规草案4件，对政府制发的72件涉法文件进行了前置合法性审查，做到所有规范文件均由法制办审核。

　　三是加强诚信政府建设。扩大公开范围，改版政府门户网站，市区两级预决算和“三公”经费支出全部向社会公开。建立政府新闻发言人制度，重大事项及时向社会发布。规范政府债务管理，维护政府金融信誉。坚持以政府诚信引领社会诚信，建立企业信用红黑榜制度，协调法检两院建立多部门联合信用惩戒机制，推动了社会诚信体系建设。

　　四是加强创新政府建设。整合工商与食药、卫生与计生、人社与公务员管理等机构，进一步理顺政府管理职能。实行供水、排水、污水处理和管网等水务一体化管理，下放城管、环卫、园林、环保事权，明确了旗县区主体责任。成立财政资金绩效管理委员会，统筹管理社保基金、住房公积金、维修基金，专项资金收益新增2亿元。

　　五是加强廉洁政府建设。严格落实党风廉政建设责任制和中央八项规定，扎紧制度笼子，着力正风肃纪，情况有了很大改观。清理超标公务用车1034台、办公用房11.2万平方米，“三公”经费同比下降12.7%，节余资金全部用于民生支出。制定加强和改进政府工作的十条规定，坚持每半年召开一次政府廉政会议，实行行政正职分管审计工作，行政监察和审计监督全面加强。

　　各位代表，过去一年，困难比预料的多，结果比预想的好，经济社会发展既有量的扩大，更有质的提升，这样的成绩来之不易。这是自治区党委、政府和市委正确领导的结果，是人大、政协和社会各界理解支持的结果，是全体干部担当奉献的结果，是广大市民共同奋斗的结果。实践证明，只要我们凝心聚力、真抓实干，就没有闯不过去的难关;只要我们主动作为、敢于担当，就一定能够实现既定的目标。这里，我们还要特别为包头的企业家点赞，正是他们对市场的信心、对事业的执着，才使我们同舟共济、共度难关。借此机会，我代表市政府一并表示深深的谢意!

　　过去一年的工作，我们也有许多不尽如人意的地方。比如，对支持中小微企业出台了很多政策，但实际执行的还不够到位;城市路网和公共交通相对滞后，老百姓出行还不够便捷;老旧小区的基础设施老化和脏乱差问题比较突出，群众思改盼改的愿望还很强烈;空气质量、食品安全、教育医疗等热点问题，还没有得到根本解决;城市城乡二元结构依然明显，一些困难群众的生活还很艰苦。这些问题，有的是发展过程中产生的，有的是工作不到位造成的。对存在的问题，政府要首先从自身找原因、想办法，切实加以解决，民之所望是我们的施政所向。

2015年总体部署

　　科学认识形势，准确把握走势，是我们做好工作的前提基础。当前，我国经济发展进入新常态，这是以习近平同志为总书记的党中央作出的重大战略判断。认识新常态、适应新常态、引领新常态，是今后一个时期经济发展的大逻辑。我们只有观念上适应，认识上到位，方法上对头，才能更好地把握工作的主动权，推动经济社会平稳健康发展。

　　一是把握新常态，要坚持抓好发展这个第一要务。新常态不是不要发展速度，而是要有合理的发展速度，必须坚定信心、坚持发展。2013年以来，我市经济增长从高速进入中高速阶段，这是宏观经济增长速度换挡期、结构调整阵痛期、前期政策刺激消化期“三期叠加”和我市自身产业结构矛盾共同作用下的必然结果，应当理性对待，保持“平常心”。但要看到，经济发展进入新常态，没有改变我国发展仍处于可以大有作为的重要战略机遇期的判断，没有改变我国经济发展总体向好的基本面。我市属于国家呼包银榆经济区重点城市，有资源、区位、成本、政策等多方面优势，有雄厚的产业基础和齐全的产业门类，新世纪以来的发展已经奠定了坚实的物质基础，实现经济增速高于全国、全区平均水平，有条件、有优势、有潜力。我们要牢牢把握这一发展大势，主动作为，积极进取，不断推动经济发展迈上新台阶。

　　二是把握新常态，要加快推进产业结构调整。新常态下的发展，不是过去那种粗放的大干快干，而是有质量、有效益、可持续的发展。近年来，我市钢铁、电力、铝业、稀土、装备制造几大主导产业一直面临着全国产能过剩、产品价格大幅下跌等问题，产业转型升级的压力持续增大。但通过大力改造提升传统产业和培育发展新兴产业，一批高耗能、高污染、生产工艺落后的企业被坚决关闭，一批带动能力强的重大项目和新技术、新产品成功引进，产业由中低端向中高端转换步伐明显加快。我们要持续发力，坚持调整存量与做优增量并重，加快推进传统产业新型化、新兴产业规模化、支柱产业多元化，不断强化节能减排和生态环境建设，着力打造包头经济升级版，推动包头经济这艘大船行稳致远。

　　三是把握新常态，要充分发挥投资关键作用。新常态下，随着我市加快打造区域消费中心、物流中心、金融中心、文化旅游中心和口岸支点，消费、出口贡献率将持续增加，但今后相当长时期投资仍是拉动我市经济增长的主要动力。我市属于欠发达地区，公共服务设施薄弱，棚户区、城中村大量存在、产业发展还属于中低端水平，水、电、交通等基础设施建设依然滞后，实现经济转型升级，必然需要大量的基础设施和产业项目投资。国家扩大预算内投资规模，在中西部地区开工建设一批重大交通、水利项目，加强大气污染治理，加快推进“一带一路”和京津冀协同发展，这对促进我市基础设施建设、清洁能源基地建设、扩大对外开放、承接产业转移等都是难得机遇。我们要放眼全局，主动融入环渤海地区发展，深化与蒙古国合作，下功夫扩投资上项目，科学审慎编制“十三五”规划，在积极服务国家发展战略中拓展发展空间，构筑新的发展优势。

　　四是把握新常态，要不断增强创新驱动能力。推动经济增长逐步由投资驱动、要素驱动向创新驱动转变，是适应经济发展新常态的必然要求。近年来，我们一直大力推进产学研一体化，引进知名院校在我市设立研发机构，加快科技成果转化，高铁用轨、大口径无缝钢管、大吨位矿用车等一批产品研发成功，稀土新材料基地正在加快建设，创新驱动作用越来越大，新的增长点不断涌现。特别是去年以来，通过大力推进简政放权，放开政府这只看得见的手，用好市场这只看不见的手，市场活力进一步释放。我们要继续依托科技创新和改革创新，加快完善现代市场体系，全面优化发展环境，推动增长动力更为多元，形成经济发展的长久支撑。

　　基于以上分析判断，我们提出2015年政府工作的总体要求是：深入贯彻党的十八大、十八届三中、四中全会精神和中央经济工作会议精神，全面落实自治区九届十三次全委会议暨全区经济工作会议和市委十一届七次全委会议决策部署，坚持稳中求进工作总基调，主动适应经济发展新常态，紧紧围绕自治区“8337”发展思路和市委"5421"战略定位，突出抓好重大项目策划实施，加快推进工业转型升级，大力发展现代服务业和农牧业，全面提升城乡建设水平，强化改革创新，强化法治建设，强化民生保障，推动经济社会平稳健康发展，为“十三五”全面建成小康社会奠定坚实基础。

　　今年经济社会发展的主要预期目标是：地区生产总值增长8%，固定资产投资增长13%，一般公共预算收入增长7%，城镇居民人均可支配收入增长9%，农村牧区居民人均可支配收入增长10%，新增城镇就业4.1万人，居民消费价格涨幅控制在3%以内。

　　2015年重点工作

　　政府工作千头万绪，但必须把握关键、突出重点。今年我们要重点抓好以下六个方面：

　　一、保持经济平稳增长

　　一是保障企业正常运行。加强对经济运行的分析研判和监测调度，适时出台经济扶持政策，针对困难企业制定“一企一策”、“一类一策”解决方案，减少亏损、停产企业数量。落实国家、自治区减税降费等政策，做好电力多边交易和大用户直供政策配套工作，减轻企业负担。继续举办银企对接会，创新信贷方式，加强对企业特别是中小企业的融资贷款支持。鼓励钢铁、有色、装备制造企业加强上下游产业协作，推进用电企业与电厂兼并重组，提升产业间的依存度和企业抗风险能力。加强煤电油气运保障，引导企业降低生产成本，调整产品结构，扩大市场份额。

　　二是实施重大产业项目。围绕建设“五个基地”，加快实施神华煤制烯烃二期、包钢稀土钢冷轧、包铝50万吨高品质铝合金、比亚迪新能源矿用车等重点项目，策划实施明拓140万吨铁素体不锈钢、包钢煤焦油深加工、高铁动车铝合金铸件等重点项目，力争中德装备制造产业园、国储能煤基多联产、鸿达兴业稀土助剂等项目年内开工建设。支持工业园区加快污水处理等基础设施建设，策划引进一批单体规模大、产业带动强的大项目。支持金属深加工和铝业深加工两个园区尽快进入千亿园区行列，力争土右旗、九原区两个工业园区产值分别突破300亿元。今年我们将实施亿元以上重点项目733个，确保年内投资突破2000亿元。

　　三是抓好六大通道建设。畅通电力、航空、陆路、燃气、口岸和数据六大通道，实施总投资722亿元的一批重大基础工程。电力通道方面，积极开展白彦花煤田总体开发等煤电项目前期工作，争取达茂旗1800万千瓦清洁能源基地和土右旗600万千瓦火电基地列入国家电力外送通道“十三五”规划。航空通道方面，依托机场旧航站楼改造申报国际口岸机场，规划建设包头空港物流园区，在达茂旗和石拐区建设两个通用机场。陆路通道方面，抓紧实施陶准铁路、金山工业园区铁路专线和呼包银榆客运专线建设，做好包头西安海口、呼市包头银川高铁列入国家“十三五”规划的前期工作。提早谋划省道224线固阳明登至土右明沙淖等7个公路项目，开工建设固阳至武川、东河至托县、美岱召至将军尧渡口和山北旅游公路，确保南绕城、省道104线希拉穆仁至百灵庙、固阳至红泥井公路建成通车。燃气通道方面，新增达旗天然气管道包头出口，为城市发展提供10亿立方米清洁能源保障，直接形成35亿元销售收入并带动天然气化工产业发展。口岸通道方面，实施包满铁路三期建设工程，推动蒙古国塔奔陶乐盖至满都拉口岸和赛音山达至满都拉口岸公路建设，推动中蒙跨国铁路和跨境经贸合作区早日实施，提早谋划自贸区建设。数据通道方面，扩大光纤宽带和无线网络覆盖范围，加快苏宁大数据西北中心、敕勒川科技数据中心、包头云计算中心等项目建设和推广应用，搭建统一的信息资源共享和管理服务平台，全面推进智慧包头建设。

　　二、加快产业转型升级

　　一是加快工业改造提升。钢铁产业要加大企业兼并重组力度，全面淘汰落后产能，发展稀土汽车板材、高强度不锈钢和高端装备制造用钢等高附加值产品，优质钢和特种钢比重达到80%以上。铝业要通过产能置换壮大规模，大力发展航空航天铝材、新型列车车体和轻量化铝型材，电解铝就地转化率达到70%。装备制造业要加强机床数字化改造，打造汽车、铁路装备、工程机械、特种钢延伸加工和煤化工装备等产业集群，做大高端装备制造业规模。稀土产业要推动资源整合和行业兼并重组，壮大北方稀土产业集团，加快发展稀土磁材深加工及应用，推进稀土合金及铌钪钍综合利用。煤化工产业要延伸煤基、化基产业链条，发展焦炉煤气制甲醇、乙二醇及焦油深加工制农药、医药中间体等精细化工产品。能源产业要解决“弃风窝电”问题，提高风光电等清洁能源装机容量。支持新能源汽车推广应用，加快发展节能环保产业。进一步加大战略性新兴产业培育力度，力争占工业比重提高到18%。

　　二是提高现代服务业水平。大力发展现代物流业，整合110、210国道和建设路两侧物流企业，推动物流企业集中融合发展。重点抓好钢铁建材物流、铁路现代综合物流和煤炭物流园区建设，力争钢铁物流和煤炭物流交易额突破250亿元和600亿元。规划建设全市统一的汽车销售和二手车交易市场，争取实施国家城市共同配送项目。大力发展金融业，加快推进钢铁大街沿线金融商务区建设，引进民生银行、渣打银行和渤海银行设立分支机构。大力发展文化旅游业，加快推进33个文化旅游景区建设。重点完善五当召、美岱召景区基础设施，全力创建5A级景区。全面开展希拉穆仁、九峰山景区环境治理，实施赛汗塔拉城中草原提档升级工程。加强与京津冀和宁陕晋地区的旅游合作，开发旅游精品线路，让更多来包头的人做兴致盎然的游客，不当匆匆而归的过客。大力培育新兴服务业，制定加快发展电子商务政策，创建国家电子商务示范城市。办好中俄蒙国际论坛、国际装备制造博览会等大型会展活动。大力提升传统商贸服务业，推进商贸集聚区、专业市场和特色商业街建设，恢复一批老字号、老品牌。实施苏宁广场、茂业天地、北京华联购物中心等99个亿元以上商贸重点项目，力争完成投资338亿元。

　　三是打造现代农牧业强市。包头不是农牧业大市，但要成为农牧业强市。大力实施“南菜北薯，乳肉并举”发展战略，加快建设现代农牧业示范园区，争取九原园区晋升国家级产业化示范基地，推进达茂旗、固阳县园区创建自治区级产业化示范园区。规划建设东河区蔬菜产业示范基地，打造现代农牧业及都市休闲示范基地30个，培育国家、自治区级龙头企业10个。鼓励注册农畜产品商标，“三品一标”产品新增60个，达到330个，举办第二届中国(国际)牛羊肉产业大会和全区绿色农畜产品博览会。

　　四是增强科技创新驱动能力。抓好北大包头创新研究院、浙大包头工业技术研究院、中科院包头稀土新材料研发中心建设，推进上海交通大学材料学院尽快落户我市。支持北重、一机等15个重大科技项目，加快铝产品深加工、煤制烯烃深加工等中试基地建设，完成北大包头创新研究院10项以上科技成果转化。加强农业科技创新，打造中科院和中国农大2个产学研示范基地。建立全市科技创新服务中心，开展国家可持续发展实验区创建工作，加快建设全国质量强市示范城市。推进人才强市工程，力争培养一批创新团队、领军人才和学术技术带头人，做好本土科研机构扶持和人才培养工作。

　　三、推动重点领域改革

　　一是推进经济体制改革。支持包钢、一机、北重、北奔等大型国有企业引进社会资本，发展混合所有制经济。推进国有企业主辅分离和辅业改制，推动企业整体上市或核心业务资产上市。支持有条件的民营企业建立现代企业制度，鼓励非公企业到资本市场直接融资。鼓励非公企业进入基础产业、基础设施、市政公用事业等领域。深入开展中小企业小额贷款履约保险工作，建立企业异常名录，深化社会诚信体系建设。推进预算管理制度改革，落实税制改革政策，做好建立事权和支出责任相适应制度的基础工作。

　　二是推进行政体制改革。做好国务院、自治区下放行政审批事项的承接，继续取消、下放和委托旗县区管理一批行政审批项目。建立市县乡三级网上办事大厅，推行网上审批。全面推行"先照后证、宽进严管"，加强对市场主体的事中事后监管。完善公共资源交易管理体制，进一步规范政府投资项目招投标和政府采购行为。在教育医疗、养老助残、社会救助等公共领域，扩大政府购买服务试点。组建市住房建设集团和国有资产投资公司，申请发行棚户区改造收益债券。

　　三是推进城镇化和农村牧区改革。有序放开落户限制，建立城乡统一的户口登记制度。稳步开展不动产统一登记，完善现代产权管理制度。完成农村土地和牧区草原确权登记颁证工作，探索土地征收、集体经营性建设用地入市和宅基地制度改革，加快土地向新型经营主体流转。实施新农村新牧区现代流通网络建设工程，组建市供销集团。

　　四是推进生态文明制度改革。开展领导干部自然资源资产责任审计试点，实施主要污染物排污权有偿使用和交易试点。开展节能和碳排放权交易试点，制定碳排放权交易管理暂行办法，创建国家低碳工业绿色发展试点城市。深化水务一体化管理改革，抓好黄河水权转让，完善水资源管理制度。建立大青山自然保护区腹地工矿企业退出机制。加强草原、森林、河流、湿地生态保护，推进生态管理机制创新。

　　五是推进社会事业改革发展。试行中小学校长职级制改革，全面推行义务教育学校校长、教师交流制度。推动青山区、达茂旗等7个旗县区通过国家县域义务教育均衡发展验收。建立国有文化资产监管机构，推动政府部门由办文化向管文化转变，完善文化市场准入退出机制和中小微文化企业发展政策，推进文化事业单位法人治理结构改革试点。深化公立医院改革，鼓励民营资本进入医疗市场，逐步建立公立医院与民营卫生机构分工协作机制。实行行业协会社会组织直接登记和备案制度，完善社会治理体制。完善养老服务经营模式，支持社会力量参与养老、助老项目，构建公办和民办相结合的养老服务模式。

　　四、促进城乡区域协调发展

　　一是增强城市服务功能。加强新都市区建设，完成综合管廊、市民公园、道路主干网等基础设施和包九中分校建设，启动文化中心和博物馆等项目前期工作，力争城市展厅和三级甲等综合医院开工建设，推动万达、苏宁、华发、绿地等知名地产企业入驻新区。完成北梁腾空区控制性详细规划，启动实施公园广场、西口古民居街区及相关配套设施建设。加强城市基础设施建设，新建改造40公里供热干线、35公里天然气管线和17.7公里污水管网，续建九原、万水泉污水处理厂，新建餐厨垃圾处理厂、污泥处理厂。启动城市轨道交通项目前期工作，新建改造沼潭公园、昆都仑召公园、井坪公园、莫尼林带公园等一批公园广场，治理昆河下游、二道沙河、四道沙河、北郊截洪沟等河道，规划建设环城景观水系。

　　二是提高县域经济发展水平。制定山北地区产业发展整体规划和协同发展的指导意见，打造山北地区经济发展带。推动白云、达茂、固阳协调发展，统筹编制"十三五"规划，加大资源勘查力度，共同建设国家级清洁能源输出基地、原材料生产加工基地和对蒙合作开放基地。加快推进土右旗中等城市建设，抓好“扩权强县”试点改革，推进以新型能源和煤化工为主的产业发展，成为全市新的经济增长极。启动黄河堤防二期、塔令宫水库、五当召水库项目，抓好小型水利建设和抗旱调水、节水灌溉工程。按照城市的标准推进萨拉齐、百灵庙、金山等城关镇建设，打造精品县城，培育特色中心集镇，促进农牧业转移人口就近实现市民化。

　　三是强化生态建设和环境保护。完成国家重点林业工程38万亩、六大重点区域绿化4.3万亩，启动实施大青山南坡生态环境综合整治工程，打造优美的城市后花园。全面完成公路铁路沿线及城市出入口环境综合整治，建立苗木管护长效机制，解决好关停企业搬迁及转型发展问题。加强北部草原生态保护，加快建设包头黄河国家湿地公园。抓好东河区铝业园区国家城市矿产示范基地和白云矿区矿产资源综合利用示范基地建设，打造循环经济新亮点。实施节能减排财政政策综合示范市项目20个，确保单位GDP能耗降低率超额完成“十二五”规划目标25%以上。高起点高标准实施好与环保部签订的《包头市环境保护整体解决方案》，治理环境突出问题，创建全国生态文明示范城市。

　　五、做好民生重点工作

　　一是大力实施创业就业工程。加大政府购买基层公共管理和社会服务岗位力度，开发公益岗位2246个。建设公共实训基地，培养技能型实用人才。发放小额担保贷款3.8亿元，扶持高校毕业生自主创业，实现就业1.7万人以上。我们要努力让群众实现就业的机会更充分一些。

　　二是大力实施百姓安居工程。加快北梁棚改3.1万户安置房建设，确保年内所有搬迁居民入住，全面完成北梁棚改任务。改造其他地区城市棚户区2.49万户，改造70个老旧小区，回购建设12个便民蔬菜市场，实现城五区物业服务全覆盖。我们要努力让群众居住的环境更舒心一些。

　　三是大力实施农村牧区“十个全覆盖”工程。投资16.8亿元，建设高标准示范村51个、达标村157个，生态脆弱地区移民4030人，逐步改善农牧民生产生活环境。我们要努力让农牧民的日子更红火一些。

　　四是大力实施精准扶贫“三到村三到户”攻坚工程。强化村企合作、股份合作及杠杆式金融扶贫等措施，力争落实各类扶贫资金8.9亿元，解决3.5万贫困人口的脱贫问题。我们要努力让困难群众的心灵更温暖一些。

　　五是大力实施城市管理提升工程。建立城市综合管理委员会，启动数字化城管指挥系统。春节后开展城市环境综合整治百日攻坚战，重点整治私搭乱建、破门开店、三轮车违法运营和部分地段脏乱差等问题，打造文化路、黄河大街等22条景观示范街。我们要努力让群众生活的环境更养眼一些。

　　六是大力实施平安包头建设工程。加强公安派出所和信息化建设，续建6个公路治安检查站，建成全市视频监控联网调度平台，抓好老旧小区的视频监控建设。完成市强戒所和特殊违法犯罪人员治疗中心建设，严厉打击涉毒等各类违法犯罪。全面加强城市应急管理，有效防范和应对公共卫生、自然灾害等各类突发性事件。我们要努力让群众的心里更踏实一些。

　　七是继续实施大气污染防治工程。实施32项大气污染治理工程，全部完成电力行业脱硫脱硝和钢铁行业脱硫改造，治理城区剩余794台燃煤锅炉和103台工业燃煤窑炉，制定实施工业与民用煤使用标准和管理办法，推广替代型煤40万吨，淘汰黄标车及老旧车辆2.6万辆，建成覆盖全市的大气监控预警系统。我们要努力让群众看到蓝天白云的日子更多一些。

　　八是继续实施供水改造工程。新建供水管网266公里，完成磴口净水厂提标改造。全部封停剩余的100多眼企业自备井，强制工业园区使用黄河水或中水。新增健康水受益人口21万，基本实现城区健康水全覆盖，新增农村牧区安全饮水受益人口1.5万。我们要努力让群众的饮水更健康一些。

　　九是继续实施食品安全监管工程。加快建设九原区、土右旗大型食品加工园区，力争引进企业20家。新建农畜产品质量追溯体系试点100个，建立综合性食品检验中心，健全餐饮食品信息化监管平台，对校园周边流动商贩食品安全问题进行专项整治。加大食品药品执法检查力度，实行网格化管理。我们要努力让群众的"舌尖"更安全一些。

　　十是继续实施交通设施改善工程。适时启动110国道、沼南大道等道路建设，完成哈屯高勒路西延过昆河桥等5座桥梁工程，打通40条城区断头路。实施建设路和巴彦塔拉大街提档升级，建设百里绿道、百里公交专线，打造从包钢厂区到东兴出口独具特色的"百里长街"。购置新能源公交车200台，新开改造5条公交线路。规划综合客运枢纽站场，开工建设青山区和固阳县长途客运站。我们要努力让群众的出行更便捷一些。

　　各位代表，民为邦本，本固邦宁。我们要满怀热情地做好民生工作，努力为人民群众创造更多的福祉。强化企业工资集体协商和工资正常增长机制，提高机关事业单位工资水平。稳步提高养老、医疗等社会保障标准，加快建设民政综合福利园区。继续实施基础教育学校标准化建设和农村牧区薄弱学校改造，新建扩建37所公办幼儿园。规划建设国家(北方)青少年足球夏令营活动基地，推动足球运动普及与提高。完成全市四级公共文化服务基础设施标准化建设工程，确保通过国家公共文化服务体系示范区验收评估。推进蒙中医院、儿童医院、妇幼保健院、市第八医院危楼改扩建等项目建设，增强公共卫生服务能力。全面落实安全生产责任制，加大隐患排查和整治力度，坚决遏制重特大安全事故发生，把人命关天的大事抓严抓实。

　　六、全面加强法治建设

　　一是依法全面履行政府职能。用法治思维和法治方式履行职责，自觉接受人大、政协及社会各界的监督，认真听取人大代表、政协委员和各民主党派、工商联、无党派人士意见。积极配合人大立法工作，支持政协开展民主协商，支持法检两院依法独立公正行使职权。健全依法决策机制，坚持重大行政决策事先征求各方面意见建议。加强重大行政决策社会稳定风险评估，完善重大行政决策合法性审查和集体讨论决定程序。完善政府法律顾问制度。健全政府权力行使的内外监督制约体系，加强对财政资金分配使用、公共资源转让、公共工程建设等权力集中部门的监督管理。全面推行权力清单、负面清单和责任清单制度，加大行政问责力度。

　　二是切实提高行政执法水平。创新执法体制，完成对工商与食药监、文化与广播电影电视、卫生与计生等领域执法力量整合，依法理顺市容环境卫生管理、违章建筑查处等方面执法职权，解决交叉执法、多头执法和不执法、乱执法问题。调整环保执法体制，完善环境监管执法稽查网络，加强基层环保力量建设。依法加大对食品药品安全、生态环境、安全生产等重点领域的执法力度。完善执法程序，建立重大执法决定审查和执法全过程记录制度。落实行政处罚裁量权基准制度，实行行政执法责任制。加强仲裁监管，实行案件问责制。加强行政复议委员会建设，提高行政复议化解行政争议的质量和效率。

　　三是深入开展法治社会建设。推动开展以宪法为核心的全民法制宣传教育，切实增强公民法治观念和法律素养。落实“谁执法谁普法”的普法责任制，扎实做好“六五”普法验收工作。推进覆盖城乡的法律服务体系建设，扩大法律援助范围。全面推行阳光信访，推动信访纳入法治化轨道。强化社会治安综合治理，有效防范化解影响社会安定的问题。认真落实党的民族宗教政策，促进民族团结、宗教和谐，依法妥善处置涉及民族、宗教等因素的社会问题。牢固树立国防意识，支持军队建设，深入推进军民融合发展，争创全国双拥模范城八连冠。

　　四是树立政府良好形象。我们将以建设法治政府为统领，持续推进诚信政府、创新政府、廉洁政府建设，严格落实党风廉政建设责任制，深入推进反腐倡廉，始终坚持从严治政，坚决执行中央八项规定、自治区和我市配套规定，毫不动摇转变作风，切实增强执行力和落实力，勤勉尽责，廉洁奉公，言必行、行必果，以改革发展的实效取信于民。

　　各位代表，时代赋予使命，奋斗创造未来。正如习近平主席新年贺词所讲，“开弓没有回头箭、改革关头勇者胜”。我们将竭尽所能、全力以赴，努力让包头的产业做得一年比一年强，城市建得一年比一年美，老百姓的日子过得一年比一年好。
